# Chondral/Desmal Osteogenesis in 3D Spheroids Sensitized by Psychostimulants

**DOI:** 10.3390/jcm11206218

**Published:** 2022-10-21

**Authors:** Nele Wagener, Wolfgang Lehmann, Kai O. Böker, Eric Röhner, Pietro Di Fazio

**Affiliations:** 1 Department of Trauma Surgery, Orthopedics and Plastic Surgery, University Medical Center Goettingen, Robert-Koch-Str. 40, 37099 Göttingen, Germany; 2 Center for Musculoskeletal Surgery, Corporate Member of Freie Universität Berlin and Humboldt-Universität zu Berlin, Charité–Universitätsmedizin Berlin, Charitéplatz 1, 10117 Berlin, Germany; 3 Orthopaedic Department, Heinrich-Braun-Hospital Zwickau, 08060 Zwickau, Germany; 4 Department of Visceral Thoracic and Vascular Surgery, Philipps University Marburg, Baldingerstraße, 35043 Marburg, Germany

**Keywords:** bone defect, cell spheroids ADHD, hMSCs, osteogenic/chondrogenic differentiation, cell viability

## Abstract

Attention deficit hyperactivity disorder (ADHD) affects 6.4 million children in the United States of America. Children and adolescents, the main consumers of ADHD medication, are in the bone growth phase, which extends over a period of up to two decades. Thus, impaired proliferation and maturation of chondrocytes and osteoblasts can result in impaired bone formation. The aim of this study is to investigate, for the first time, the effects of the ADHD-medication modafinil, atomoxetine and guanfacine on bone growth and repair in vitro. Using two-dimensional and three-dimensional cell models, we investigated the chondrogenic/osteogenic differentiation, proliferation and viability of human mesenchymal progenitor cells. Real-time cell proliferation was measured by xCELLigence. Live/dead staining and size measurement of hMSC- and MG63 monolayer and spheroids were performed after administration of therapeutic plasma concentrations of modafinil, atomoxetine and guanfacine. Chondrogenic differentiation was quantified by RTqPCR. The chondrogenic and osteogenic differentiation was evaluated by histological cryo-sections. Modafinil, atomoxetine and guanfacine reduced chondrogenic and osteogenic differentiation terms of transcript expression and at the histological level. Cell viability of the MG63- and hMSC monolayer was not impeded by ADHD-medication. Our in vitro results indicate that modafinil, atomoxetine and guanfacine may impair chondrogenic and osteogenic differentiation in a 3D model reflecting the in vivo physiologic condition.

## 1. Introduction

Attention deficit disorder (ADHD) from childhood to adulthood is a syndrome that affects millions of people and is often associated with lifelong medication [1]. The three psychostimulants modafinil, atomoxetine and guanfacine are normally prescribed for the treatment of ADHD and are also increasingly abused for performance enhancement purposes. In particular, children and adolescents may be the most affected as they are in the process of bone formation [1].

Growth and repair of bone occurs primarily from within using a cartilaginous template (enchondral ossification), starting with mesenchymal cell groups [2]. Proliferating chondrocytes form a matrix rich in type II collagen and aggrecan [3,4]. Later, the mineralization of the extracellular environment begins. Most chondrocytes eventually undergo apoptosis, some develop into osteoblasts [3]. Enchondral ossification, which spans two decades, can be impaired due to the impaired proliferation and maturation of chondrocytes in the growth plates, leading to shortened length growth and impaired fracture healing [3,5,6].

Studies from 2018 confirm an increased incidence of stress fractures when taking an NA-dopamine-reuptake inhibitor, leading to bone imbalance and fracture susceptibility [7]. Another study involving 2400 soldiers showed that the methylphenidate based therapy in ADHD patients caused a decrease of the bone density, which correlated with a higher rate of stress fractures [7,8]. Ching Chou et al. demonstrated that fracture rates were positively correlated with ADHD diagnosis in a study cohort of 3640 children [9]. As a first step, with this research we would like to investigate the influence of ADHD medication on the genes AGGRECAN, SOX9 and COL2A1, which are the genes that are most related to chondrogenic differentiation. Thus, the chondrogenic differentiation state of the hMSC included in this study is strictly related to the expression of chondrogenic differentiation markers. SOX9, the master transcription factor, is essential for chondrocyte differentiation and cartilage homeostasis. It is a transcription factor that is significantly involved in enchondral ossification through condensation and aggregation of undifferentiated MSC to differentiate into mature and hypertrophic chondrocytes [10]. Aggrecan, the most represented proteoglycan in articular cartilage, is expressed by chondrocytes and has the ability to bind hyaluronan, thereby acting as a mediator between chondrocytes and their matrix [11]. Type II collagen, the protein encoded by the gene COL2A1, functions as a chondrogenic structural protein that provides structure and strength to cartilage [12]. Currently, the research findings have been limited to the antiproliferative and viability inhibitory effects of methylphenidate on primary human chondrocytes by Gumustas et al. [13].

Wagener and colleagues were recently able to show that modafinil, atomoxetine, and guanfacine inhibited hMSC osteogenic differentiation and migration without inducing apoptosis or necrosis. The migration inhibitory effect of these psychostimulants could be abolished by the administration of the selective beta-2 blocker ICI-118551 [14]. For the first time, this research will investigate the effects of the three psychostimulants on chondrogenic and osteogenic differentiation of three-dimensional hMSC and MG63 cell spheroids. Furthermore, cell proliferation after the administration of modafinil, atomoxetine and guanfacine will be explored at the two- and three-dimensional level to determine whether bone quality is affected. The aim of this study was to clarify whether there is an influence of modafinil, atomoxetine and guanfacine on the chondrogenic and osteogenic differentiation of hMSC spheroids.

## 2. Materials and Methods

### 2.1. Cell Culture

The Human mesenchymal stem cell line SCP1 expressing hTERT was cultured in DMEM medium containing low glucose, 10% fetal calf serum (FCS), and 1 U/mL antibiotics (penicillin and streptomycin). The human osteoblast MG63 cell line was obtained from CLS (Cell Line Service, Eppelheim, Germany, product, no. 300441) and was maintained in DMEM-F12 (Gibco, Paisley, UK) with the additions of 10%FCS and 1 U/mL Pen/Strep. (Gibco, Paisley, UK). Incubation of all cells was performed under standard conditions in humidified incubators at 37 °C and 5% CO_2_.

### 2.2. Substances

The ADHD medication modafinil (11.2 µg/mL), atomoxetine (0.9 µg/mL), and guanfacine (17.7 ng/mL) was ordered from Sigma-Aldrich (Steinheim, Germany) and used in max. therapeutic plasma concentration [15,16,17]. The medication of modafinil solution (1 mg/mL), atomoxetine hydrochloride solution (1 mg/mL) and guanfacine hydrochloride (500 mg) was purchased from Sigma-Aldrich (Steinheim, Germany) and dissolved in sterile DMSO. The osteogenic differentiation medium consisting of ascorbic acid-2-phosphate (200 µM, Cayman Chemical, Ann Arbor, MI, USA), dexamethasone (0.1 µM) and ß-glycerol phosphate (10 mM, Merck, Germany) was used. 10% DMSO was purchased from Merck, Germany.

### 2.3. xCELLigence Real Time Monitoring of Cell Proliferation

HMSC and MG63 monolayer cultures (50,000/well) were cultured on E-plates (OLS, Bremen, Germany) and real time proliferation was measured for 100 h after administration of the maximum therapeutic plasma concentration of modafinil (11.2 µg/mL), atomoxetine (0.9 µg/mL) and guanfacine (17.7 g/mL) by an xCELLigence RTCA DP system (Roche, Basel, Switzerland). The real-time proliferation measurement was performed by cell contacts with gold electrodes at the well bottom, which induce an increase of impedance.

### 2.4. Establishment of HMSC- and MG63-Derived Spheroids

Cultivation of hMSC and MG63 spheroids was performed with 5000 and 8000 cells each on 1.5% agarose in a flat 96-well plate for 21 days. First, the 96-well plates were incubated on an orbital shaker with a shaking speed of 40 rpm for 12–18 h under cell culture conditions. The change of medium was performed at two-day intervals with the addition of modafinil (11.2 µg/mL), atomoxetine (0.9 µg/mL) and guanfacine (17.7 ng/mL).

### 2.5. Spheroid Size Evaluation

After microphotography, the spheroid size was determined (Leica DMi8, Wetzlar, Germany) with Image J software (NIH, Bethesda, Rockville, MA, USA).

### 2.6. Chondrogenic Spheroid 3D Culture

Production of hMSC spheroids (250,000 cells/well) was achieved by centrifugation (200× *g*) for 5 min. Chondrogenic differentiation of hMSC spheroids was then performed for 21 days using chondrogenic differentiation medium (CDM, DMEM low- glucose containing 10% FCS, 1% Pen/Strep., 100 mM sodium pyruvate, 100 µM dexamethasone, 1 µg hTGF-b1, 4 g proline, 1 mL ITS and 2895 mg ascorbic-acid-2- phosphate) and the administration of modafinil (11.2 µg/mL), atomoxetine (0.9 µg/mL) and guanfacine (17.7 ng/mL) was performed at two-day intervals (Table 1).

### 2.7. Osteogenic Spheroid 3D Culture

First, hMSC spheroids (250,000/well) were prepared by centrifugation (200× *g*) for 5 min. The addition of osteogenic differentiation medium (ODM, DMEM low- glucose containing 10% FCS, 1% Pen/Strep., 200 µM ascorbic acid-2-phosphate, 0.1 µM dexamethasone and 10 mM ß-glycerolphosphate) and the administration of modafinil (11.2 µg/mL), atomoxetine (0.9 µg/mL) and guanfacine (17.7 ng/mL) was performed at two-day intervals for 28 days (Table 2).

### 2.8. RNA Isolation and Quantitative RT-PCR

HMSC-spheroids (250,000 cells/spheroid) were differentiated with the addition of chondrogenic differentiation medium (CDM) (Table 1), modafinil (11.2 µg/mL), atomoxetine (0.9 µg/mL) and guanfacine (17.7 ng/mL) for an incubation period of 21 days. RNA extraction was performed using Trizol reagent (Invitrogen, Waltham, MA, USA) according to the manufacturer’s instructions. RNA quality and concentration was measured using DS-11 FX with an integrated spectrophotometer (Thermo Fisher Scientific, Waltham, MA, USA). For quantitative analysis of mRNA, 1000 ng of RNA was reverse transcribed and amplified using the iScript^TM^cDNA Synthesis Kit (170-8891, Bio-Rad Laboratories, Hercules, CA, USA) using thermocycler (Labcycler, SensoQuest, Göttingen, Germany). Primer sequences for SOX9, Aggrecan and COL2A1 were described in the open accessible primerbank [18,19,20]. Oligonucleotides were synthesized by Microsynth AG (Balgach, Switzerland).

The primers were used for human target genes (Table 3) using the SsoAdvanced Universal SYBR Green Supermix System (BioRad Laboratories, Hercules, CA, USA) and the RT-qPCR thermocycler CFX96^TM^Real-Time System (Bio-Rad Laboratories, Hercules, CA, USA). Results were analysed with the Bio-Rad CFX Manager (Bio-Rad Laboratories) and normalized with mRNA GAPDH content for each sample. All calculations for the relative results were done using the standard 2^−∆∆CT^ method.

### 2.9. Staining of Cryosectioned Spheroids

Chondrogenic differentiated hMSC spheroids were fixed on day 21 of differentiation, whereas osteogenic differentiated hMSC spheroids and MG63 spheroids were fixed on day 28 of incubation with 3–4% formaldehyde for 72 h. After six hours of incubation with 100 µL of 15% sucrose, the spheroids were frozen on dry ice and then embedded with Tissue Tek (R1180-X, Plano GmbH, Wetzlar, Germany). Cryosections (5–10 µm) were made on the microtome at −25 °C and transferred to a slide. Chondrogenesis was detected by Alcian blue staining, whereas osteogenesis was detected by detecting mineralized areas using alizarin red. For this purpose, the slides were washed with distilled water and stained with the Alcian blue solution (1% Alcian blue 8GX (Sigma-Aldrich, Germany) in 3% acetic acid, pH) for 30 min, followed by a 5-min counterstaining with nuclear fast red solution (0.1% Nuclear Fast Red (Sigma-Aldrich, Germany) and 5% Aluminium sulfate octahydrate (Roth, Germany) in distilled water). Staining with the alizarin red solution (2% Alizarin Red S (Sigma-Aldrich, Germany) in distilled water, pH 4.1–4.3) was done for 10 min. Calcium deposits and chondrogenic areas were detected by light microscopy (Leica DMi8, Wetzlar, Germany).

### 2.10. Live/Dead Vitality Staining

Analysis of cell viability of the hMSC/MG63-monolayer was based on fluorescence-live-dead staining with fluorescein diacetate (FDA, F7378, Sigma Aldrich) and propidium iodide (PI, CN74.2, Carl Roth). Viable cells take up the non-fluorescent FDA and convert it to a green fluorescent metabolite, whereas the nuclear stain PI accumulated only in the nuclei of the dead cells. The Incubation of the monolayer cultures was done with FDA (5 mg/mL) and PI (2 mg/mL) for 5 min in serum-free medium. Analysis was performed using a fluorescence microscope (Leica DMi8, Wetzlar, Germany) with appropriate filter sets, with densitometric quantification using Image J software.

### 2.11. Software and Statistical Analysis

Significance in difference between undifferentiated, differentiated, modafinil-, atomoxe- tine- and guanfacine treated hMSC and MG63 was determined by ANOVA followed by Tukey’s post hoc tests. Data analysis was performed using Excel 2022 (Microsoft, Redmond, WA, USA) and GraphPad Prism 9 (GraphPad Software, San Diego, CA, USA). All results show means including standard deviation. Statistical significance is indicated with asterisks (* *p* < 0.05; ** *p* < 0.01; *** *p* < 0.001), * corresponds to a value of *p* < 0.05, ** corresponds to a value of *p* < 0.01, *** corresponds to a *p*-value of *p* < 0.001. All experiments were repeated with three technical and six biological replicates.

## 3. Results

### 3.1. Analysis of 2D Cell Proliferation in Modafinil, Atomoxetine and Guanfacine Treated MG63 and hMSC

**Figure 1:**
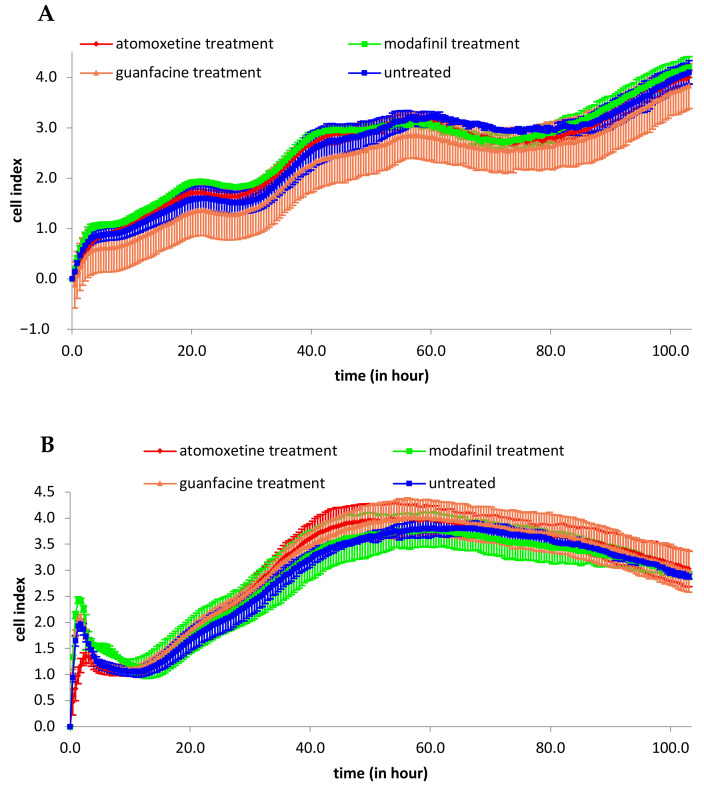
XCELLigence analysis of real-time cell proliferation of untreated MG63 (**A**), untreated hMSC (**B**) and after the administration of modafinil (11.2 μg/mL), atomoxetine (0.9 µg/mL) and guanfacine (17.7 ng/mL) to hMSC and MG63 for 100 h. Shown are means of normalized cell index ± SD of three independent experiments with six biological replicates.

The proliferation measurements of MG63 and hMSC after administration of modafinil, atomoxetine and guanfacine for a period of four days showed no effect compared to the untreated control (Figure 1A,B). Maximum proliferation of MG63 showed a cell index of 4.0 cell after 100 h of incubation. Instead, the proliferation of treated and untreated hMSC achieved a cell index of 4.0 after an incubation period of 46 h.

### 3.2. Analysis of the Spheroid Size of 5000/8000-Cell MG63 Spheroids

**Figure 2:**
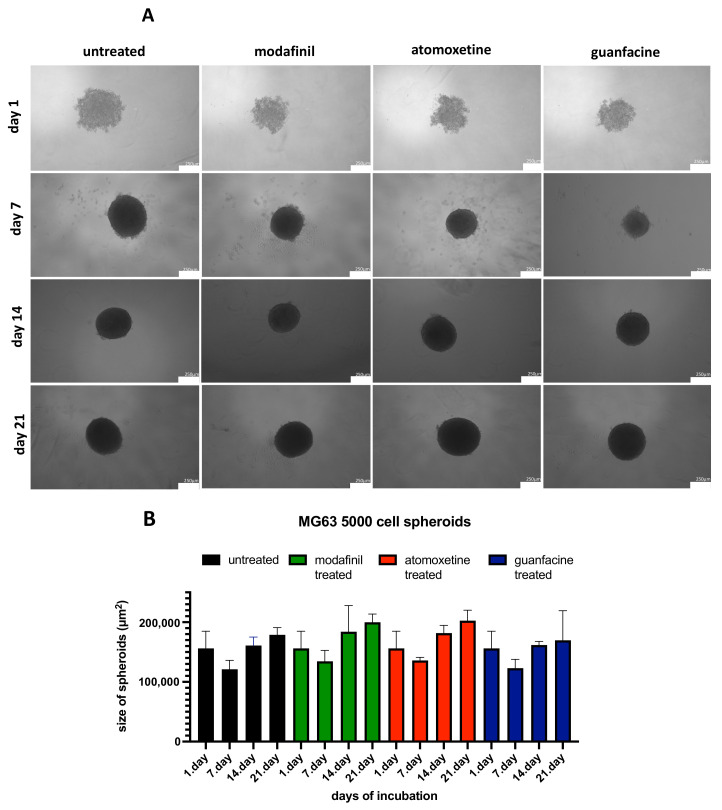

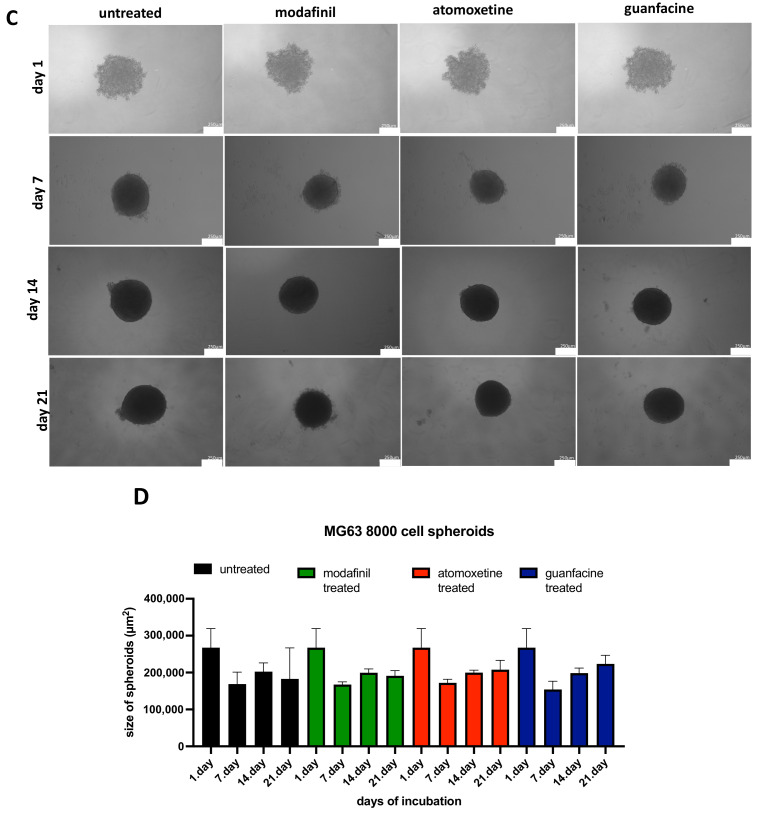
Effect of starting cell number after administration of untreated modafinil (11.2 μg/mL), atomoxetine (0.9 µg/mL) and guanfacine (17.7 ng/mL) on spheroid size and morphology of MG63 (5000 cells) (**A**,**B**) and MG63 (8000 cells) (**C**,**D**). Spheroids were cultured for 21 days. Phase- contrast images of spheroids were taken at day 7, 14 and 21. Shown are means of spheroid size ± SD of three independent experiments with six biological replicates. Densitometric quantification of spheroid size was calculated by ImageJ software. Significance in difference between the groups was determined by ANOVA followed by Tukey’s post hoc test. Scale bar 250 µm.

On the 21st day of incubation, untreated MG63 spheroids obtained with a starting cell count of 5000 and 8000 showed no significant difference in terms of spheroid size compared to modafinil-, atomoxetine- and guanfacine-treated MG63 spheroids (Figure 2A–D).

### 3.3. Analysis of the Spheroid Size of 5000/8000-cell hMSC Spheroids

**Figure 3:**
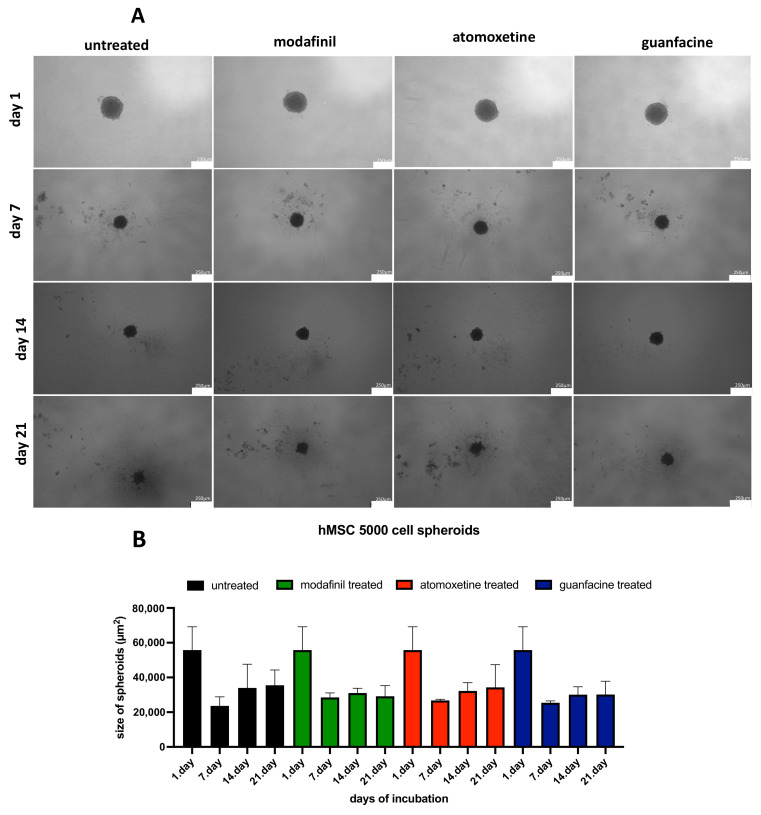

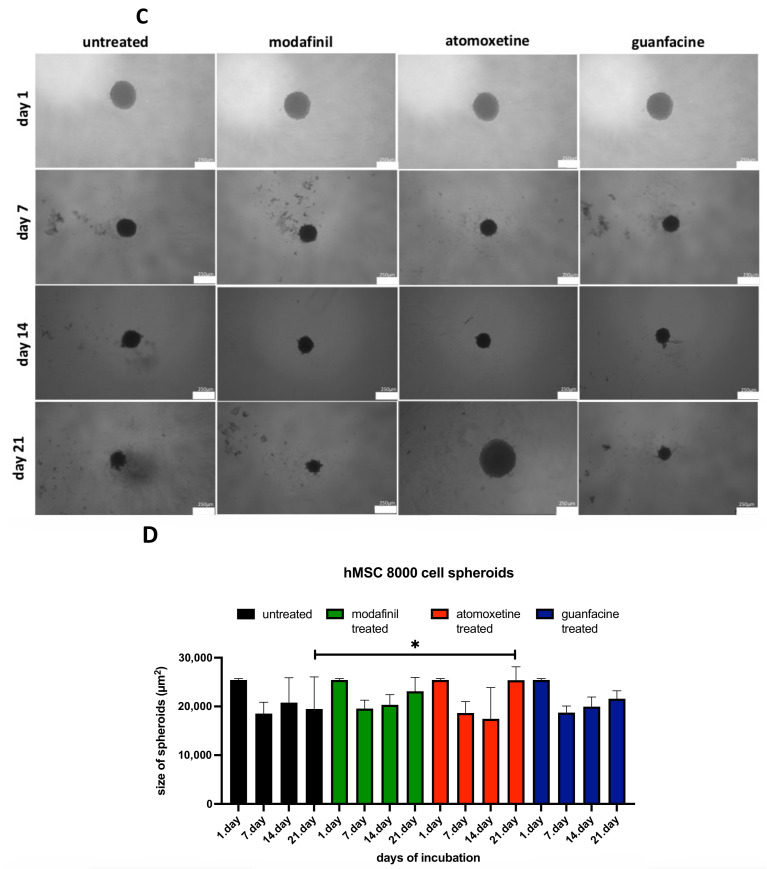
The effect of starting cell number under administration of untreated modafinil. (11.2 μg/mL), atomoxetine (0.9 µg/mL) and guanfacine (17.7 ng/mL) on spheroid size and morphology of hMSC (5000 cells) (**A**,**B**) and hMSC (8000 cells) (**C**,**D**). Spheroids were cultured for 21 days. Phase-contrast images of spheroids were taken at day 7, 14 and 21. Shown are means of spheroid size ± SD of three independent experiments with six biological replicates. Densitometric quantification of spheroid size was calculated by ImageJ software. Significance in difference between the groups was determined by ANOVA followed by Tukey’s post hoc test. Scale bar 250 µm. * corresponds to a value of *p < 0.05*.

Drug-treated hMSC spheroids showed no significant difference in terms of spheroid size compared to untreated hMSC spheroids (Figure 3A–D). Atomoxetine-treated hMSC spheroids with starting cell count of 8000 showed a significantly bigger spheroid than untreated hMSC spheroids.

### 3.4. Histological Spheroid Sections of Chondrogenic Spheroids after the Administration of Modafinil, Atomoxetine and Guanfacine

**Figure 4:**
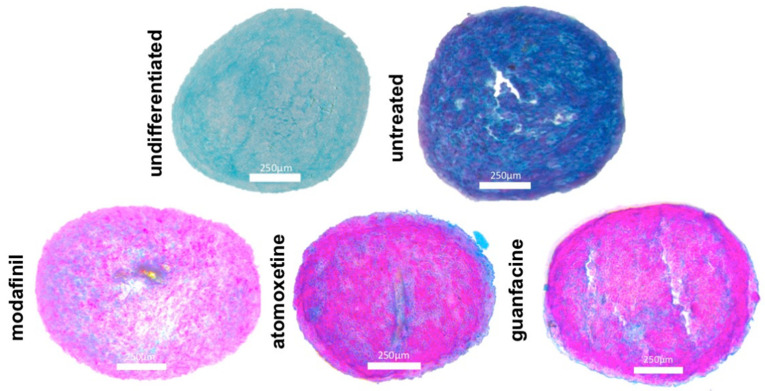
Chondrogenic differentiation of mesenchymal stem cell spheroids for 21 days. Alcian blue nuclear fast red stained images of untreated, modafinil (11.2 μg/mL)-, atomoxetine- (0.9 µg/mL) and guanfacine- (17,675 ng/mL) treated chondrogenic spheroids. Scale bar 250 µm.

Untreated chondrogenic differentiated spheroids showed blue stained acidic mucopolysaccharides, dark blue collagenous fibres and reddish appearing nuclei on day 21 after Alcian blue nuclear red staining (Figure 4). Chondrogenic differentiated spheroids (day 21) showed predominantly faint pink stained cytoplasm, pink stained nuclei and a very small amount of blue stained acidic mucopolysaccharides after the administration of modafinil (Figure 4) after Alcian blue nuclear red staining. Atomoxetine- and guanfacine treated chondrogenic spheroids (Figure 4) demonstrated a predominantly pink coloured cytoplasm with pink to red coloured nuclei, analogous to modafinil-treated chondrocyte spheroids. Alcian blue-stained acidic mucopolysaccharides occupied a slightly larger spheroid fraction compared to modafinil-treated spheroids.

### 3.5. Influence of Modafinil, Atomoxetine and Guanfacine on the Expression of SOX9, Aggrecan and COL2A1

**Figure 5:**
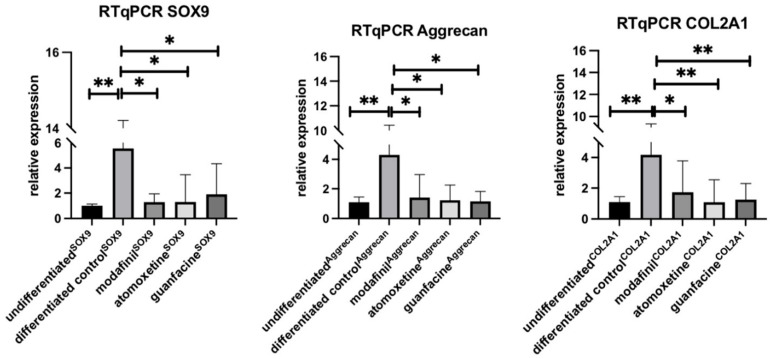
Expression of SOX9, Aggrecan and COL2A1 transcripts was determined in untreated, modafinil- (11.2 μg/mL), atomoxetine- (0.9 µg/mL) and guanfacine- (17.7 ng/mL) treated chondrogenic spheroids. Shown are means of normalized expression ± SD of three independent experiments with six biological replicates. The GAPDH transcript level was used to normalize the expression of the target genes. Significance in difference between the groups was determined by ANOVA followed by Tukey’s post hoc test. * corresponds to a value of *p* < 0.05. ** corresponds to a value of *p* < 0.01.

HMSC spheroids showed a significant over-expression of *SOX9, Aggrecan* and *COL2A1* compared to undifferentiated spheroids after 21 days of chondrogenic differentiation (Figure 5). Interestingly, the administration of modafinil, atomoxetine or guanfacine hampered the effect determined by the chondrogenic differentiation, thus causing a significant downregulation of those genes. The expression of all genes was comparable to the untreated cells.

### 3.6. Histological Spheroid Sections of Osteogenic Spheroids after Administration of Modafinil, Atomoxetine and Guanfacine

**Figure 6:**
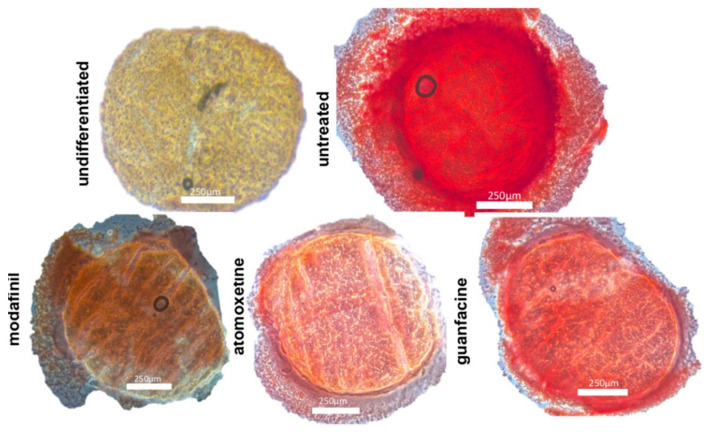
Osteogenic differentiation of mesenchymal stem cell spheroids for 28 days. Alizarin red stained images of untreated, modafinil (11.2 μg/mL)-, atomoxetine (0.9 µg/mL)- and guanfacine- (17.7 ng/mL) treated osteogenic spheroids. Three independent experiments with six biological replicates were carried out. Alizarin red (A) stained images of untreated, modafinil- (11.2 μg/mL), atomoxetine- (0.9 µg/mL) and guanfacine (17.7 ng/mL)- treated MG63 spheroids. Three independent experiments with six biological replicates were carried out. Scale bar 250 µm.

Untreated hMSC spheroids showed homogenous and intense bone-specific alizarin red staining, indicative of calcium deposition. The entire spheroid surface was strongly stained after 28 days of osteogenic differentiation (Figure 6). Atomoxetine- and guanfacine-treated hMSC spheroids displayed significantly attenuated alizarin red staining over the entire spheroid surface after 28 days of osteogenic differentiation (Figure 6). HMSC spheroids demonstrated a partially brown-stained spheroid surface after the administration of modafinil (Figure 6).

### 3.7. Histological Spheroid Sections of MG63 Spheroid after Administration of Modafinil, Atomoxetine and Guanfacine

**Figure 7:**
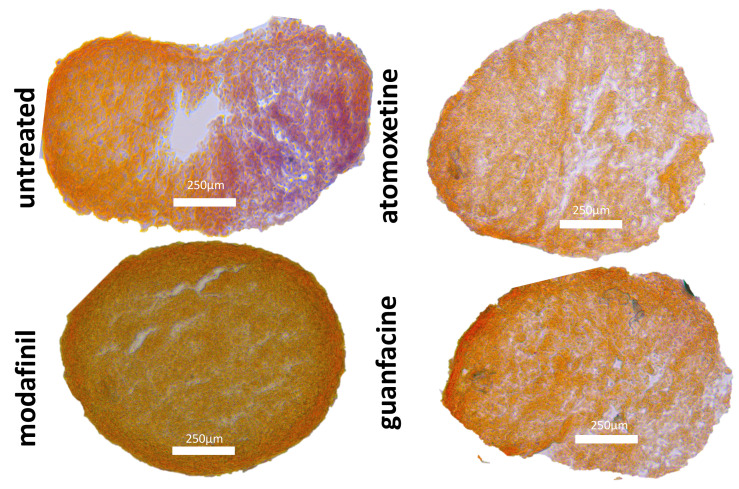
Alizarin red stained images of untreated, modafinil (11.2 μg/mL)-, atomoxetine- (0.9 µg/mL) and guanfacine- (17.7 ng/mL) treated MG63 spheroids. Three independent experiments with six biological replicates were carried out. Scale bar 250 µm.

After an incubation of 28 days, untreated, modafinil-, atomoxetine- and guanfacine- treated MG63 spheroids did not show bone-specific alizarin red staining (Figure 7).

### 3.8. Live/Dead Staining of MG63-Monolayer after Administration of Modafinil, Atomoxetine and Guanfacine

**Figure 8:**
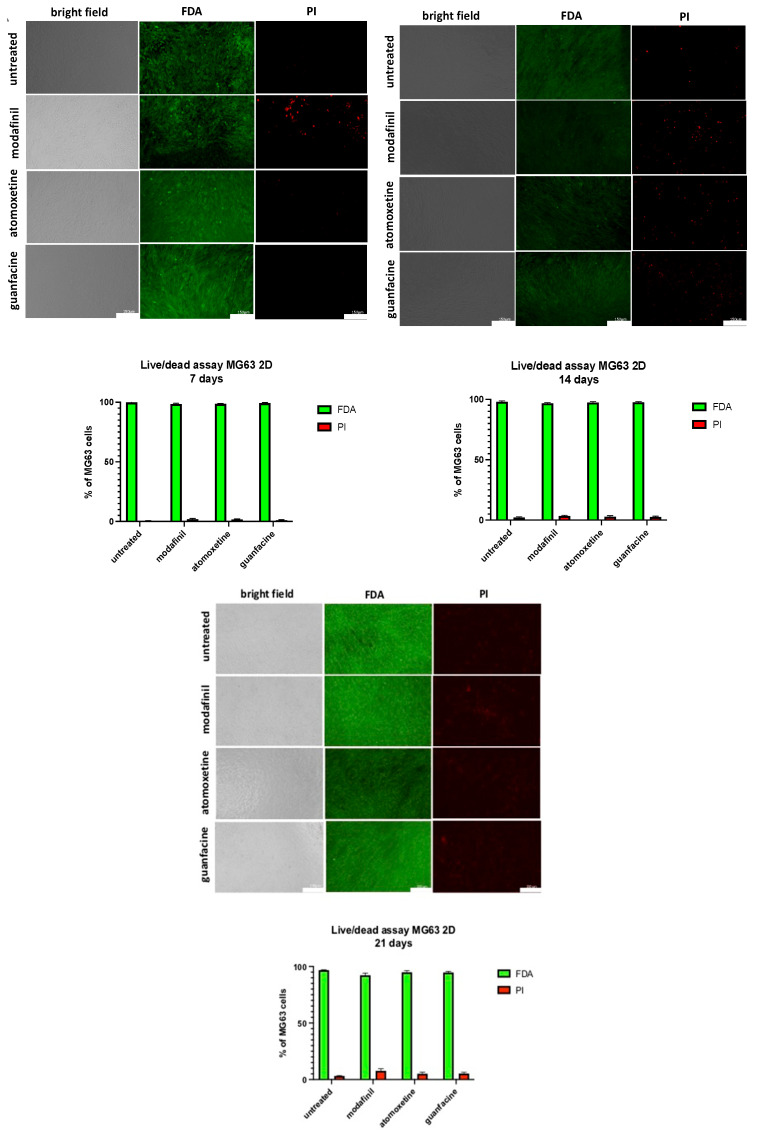
Cell viability assay of MG63-monolayer, using a live/dead staining with FDA. (green, live cells) and PI (red, dead cells). Images represent 7, 14 and 21-day-old monolayer MG63 of untreated, modafinil- (11.2 μg/mL), atomoxetine- (0.9 µg/mL) and guanfacine- (17.7 ng/mL) treated cells. Shown are three independent experiments with six biological replicates. Densitometric quantification of viable (green) and nonviable (red) cells was calculated by ImageJ software. Significance in difference between the groups was determined by ANOVA followed by Tukey’s post hoc test. Scale bar 150 µm.

On the 7th, 14th and 21st incubation day, MG63 monolayer cells, which were treated with psychostimulants, showed no significant reduction of cell viability compared to untreated MG63 (Figure 8). 

### 3.9. Live/dead Staining of hMSC- Monolayer after Administration of Modafinil, Atomoxetine and Guanfacine

**Figure 9:**
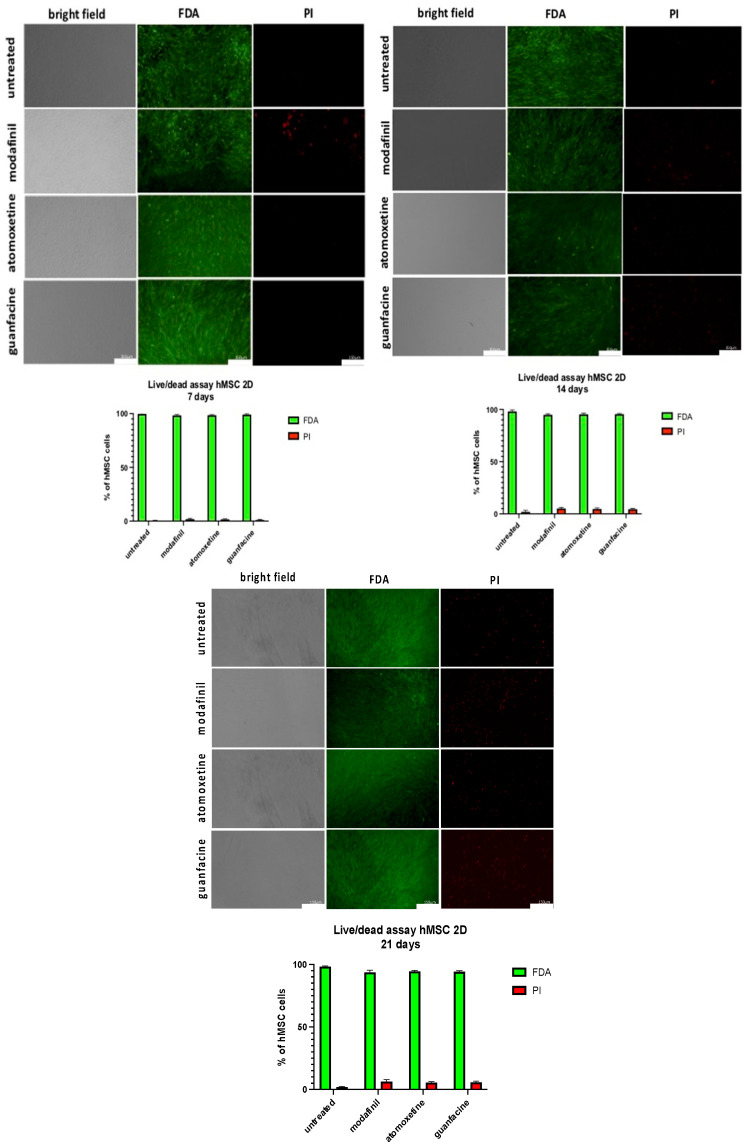
Cell viability assay of hMSC-monolayer, using a live/dead staining with FDA. (green, live cells) and PI (red, dead cells). Images represent 7, 14 and 21-day-old monolayer hMSC of untreated, modafinil- (11.2 μg/mL), atomoxetine- (0.9 µg/mL) and guanfacine- (17.7 ng/mL) treated cells. Shown are three independent experiments with six biological replicates. Densitometric quantification of viable (green) and nonviable (red) cells was calculated by ImageJ software. Significance in difference between the groups was determined by ANOVA followed by Tukey’s post hoc test. Scale bar 150 µm.

On the 7th, 14th and 21st incubation day, treated hMSC monolayer cells showed no significant reduction in cell viability compared to untreated hMSC (Figure 9).

## 4. Discussion

Our results show that modafinil, atomoxetine and guanfacine caused a reduction of the expression of the chondrogenic genes SOX9, aggrecan and COL2A1. The staining of the chondrogenic spheroid sections demonstrated a reduced Alcian blue staining which strongly correlated with the modulation of the transcripts of the chondrogenic genes.

The effect of modafinil, atomoxetine and guanfacine on osteogenic differentiation displayed a strong decalcification by a weak alizarin red staining. No effect was found in the proliferation measurement after the administration of the drugs in comparison to untreated hMSC and MG63 in the 2D and 3D models. Modafinil, atomoxetine and guanfacine caused no significant decrease of cell viability of hMSC and MG63-mono- layer on day 21 of incubation.

The transcription factor SOX9 controls the three differentiation steps during chondrogenesis, namely the aggregation and condensation of hMSC, their differentiation into mature chondrocytes and finally the hypertrophy of the chondrocytes [21]. Modafinil inhibits the three differentiation steps of chondrogenesis by the significant downregulation of SOX9, aggrecan and COL2A1 (Figure 5).

Aggrecan is an essential protein of the chondrogenic extracellular matrix that plays a key role due to its high glycosaminoglycan content and is instrumental in the creation of cartilage volume [22]. Guanfacine-treated hMSC spheroids showed significant downregulation of SOX9, aggrecan and COL2A1. Collagen type 2A1 is an essential structural protein that provides viscoelasticity to cartilage tissue [23].

The psychostimulants modafinil, atomoxetine and guanfacine significantly reduced the level of the COL2A1 transcript. In conclusion, modafinil, atomoxetine and guanfacine can significantly inhibit the expression of all three proteins involved in cartilage synthesis. Taken together, the long-term use of psychostimulants in children and adults could have a negative effect on cartilage and bone synthesis. Acute bone fractures could also result in impaired fracture healing [24].

Indicative of cellular chondrogenesis in untreated hMSC spheroids in the intense and homogenous Alcian blue staining that detects sulphated proteoglycans, glycosaminoglycans and collagen fibres in our untreated HMSC spheroids [25,26]. In contrast, our study results show that the three psychostimulants significantly inhibited chondrogenic differentiation. Clinically, a disturbed cartilage/bone metabolism could be associated with chronic intake [27]. Our study results show that the three psychostimulants can induce inhibited bone matrix mineralization through significantly reduced alizarin red staining. Insufficient bone mineralization could already be associated with early osteoporosis in children/adolescents [28].

Interestingly, after a single drug application, modafinil, atomoxetine and guanfacine showed no effect on the proliferation of monolayer MG63 and hMSC after an incubation period of four days, whereas atomoxetine, after drug application at two-day intervals, caused a significantly larger spheroid area in hMSC-spheroids on the 21st incubation day. We suspect that this result is due to the only single drug application in the monolayer cells.

To assess long-term effects of ADHD medication on bone cell viability, we cultured hMSC and MG63 monolayer for 21 days. The measurement of viability of the monolayer cells showed no significant increase of necrosis, detectable after PI staining, after incubation with modafinil, atomoxetine and guanfacine on the 7th, 14th and 21st day in both cell types.

The results show that the long-term use of psychostimulants, with drug application at two-day intervals, revealed no significant reduction of the viability after 21 days. A direct comparison with other authors is not possible due to a lack of studies, so we discuss substances with a comparable mechanism of action. Gumustas and colleagues investigated the influence of methylphenidate on the proliferation, viability and differentiation of human primary isolated chondrocytes [13].

Methylphenidate caused a reduction of cell viability and proliferation [29,30].

In contrast, we showed that no impaired proliferation of progenitor chondrocytes (monolayer hMSC) and MG63 was observed after single modafinil, atomoxetine and guanfacine administration for four days. In contrast to Gumustas et al., we detected no significant decrease in cell viability of hMSC- and MG63 monolayer cells after the administration of modafinil, atomoxetine and guanfacine in a two-day interval after 21 days [13]. Compared to Gumustus et al., we investigated the longer-term effect of administering ADHD medication in two-day intervals.

Taking into account enchondral ossification, bone health in children/adolescents in the growth phase should be closely monitored during and after long-term ADHD therapy, as disturbances in cartilage/bone metabolism cannot be ruled out [31]. Further consistent study results of the psychostimulus methylphenidate point to the reduced viability and proliferation of nucleus pulposus (NPCs) and annulus fibrosus cells (AFCs) which were isolated from 13 patients [32].

The current study is limited by the fact that the human metabolism of the ADHD drug used is not considered due to the in vitro study design. It is further limited by the short-term nature of the experiments.

With our study, we were able to show that the active substances modafinil, atomoxetine and guanfacine can have osteocatabolic effects by inhibiting osteogenic and chondrogenic differentiation [33].

As a result, disorders of bone formation, remodeling and healing could become clinically manifest.

In particular, adolescents may be affected by growth plate disorders in enchondral ossification due to limitations in longitudinal growth [34].

## 5. Conclusions

Our results showed that modafinil, atomoxetine and guanfacine could impair chondrogenic and osteogenic differentiation in vitro on a 3D level.

## Figures and Tables

**Table 1:** Chondrogenic differentiation medium.

Component Volume/Concentration Company
DMEM (low glucose) 500 mL, Sigma Aldrich, Taufkirchen, Germany
Fetal calf serum (FCS)50 mL (10%), PAN Biotech, Aidenbach, Germany
Penicillin/streptomycin5 mL (1%), PAN Biotech, Aidenbach, Germany
sodium pyruvate 100 mM, PAN Biotech, Aidenbach, Germany
dexamethasone 100µM, Carl Roth, Karlsruhe, Germany
hTGF-b10.1 mg/mL, PAN Biotech, Aidenbach, Germany
L-proline 4 g PAN Biotech, Aidenbach, Germany
ITS1 mL PAN Biotech, Aidenbach, Germany
ascorbic acid-2 phosphate 2895 mg Cayman Chemical Company, Ann Arbor, MI, USA

**Table 2:** Osteogenic differentiation medium.

Component Volume/Concentration Company
DMEM (low glucose) 500 mL, Sigma Aldrich, Taufkirchen, Germany
Fetal calf serum (FCS) 50 mL (10%), PAN Biotech, Aidenbach, Germany
Penicillin/streptomycin 5 mL (1%), PAN Biotech, Aidenbach, Germany
ascorbic acid-2 phosphate 200 µM, Cayman chemical company, Ann Arbor, MI, USA
ß-glycerolphosphate10 Mm, Carl Roth, Karlsruhe, Germany
dexamethasone 0.1 µM, Carl Roth, Karlsruhe, Germany

**Table 3:** Human primer sequences for real-time qPCR assessment.

Gene Primer Sequences
GAPDHFor: 5′- AGGTCGGAGTCAACGGAT-3′
Rev:5′-TCCTGGAAGATGGTGATG-3′
SOX9 For: 5′-AGGAAGCTCGCGGACCAGTAC-3′
Rev:5′-GGTGGTCCTTCTTGTGCTGCAC-3′
AggrecanFor: 5′- GAAAGGCATCGTGTTCCATT-3′
Rev: 5′- ACGTCCTCACACCAGGAAAC- 3′
COL2A1 For: 5′- CCTGGCAAAGATGGTGAGACAG-3′
Rev: 3′-CCTGGTTTTCCACCTTCACCTG-5′

## Data Availability

Not applicable.
